# Ultrasound-Guided Intra-Articular Infiltration of Hyaluronic Acid, Lidocaine, and Methylprednisolone in Patients with Temporomandibular Disorders (TMD): A Preliminary Pilot Case Series

**DOI:** 10.3390/clinpract16060111

**Published:** 2026-06-12

**Authors:** Giuseppe Messina, Francesco Mantia, Pietro Cataldo, Angelo Iovane

**Affiliations:** 1 Department of Human Sciences and Promotion of the Quality of Life, San Raffaele University of Rome, 00166 Rome, Italy; 2 Posturalab Research Institute, 90134 Palermo, Italy; 3 Centro Medico Mantia, 90142 Palermo, Italy; francesco@centromedicomantia.it; 4 Sport and Exercise Sciences Research Unit, Department of Psychology, Educational Science and Human Movement, University of Palermo, 90142 Palermo, Italy; pietro.cataldo@unipa.it

**Keywords:** Temporomandibular Joint (TMJ), Temporomandibular Disorders (TMD), Hyaluronic Acid, Ultrasound-Guided Infiltration, Preauricular Pain

## Abstract

Background/Objectives: This preliminary pilot case series aims to evaluate the feasibility and temporal evolution of pain and function following an ultrasound-guided infiltration technique with hyaluronic acid and methylprednisolone in a specific patient population with Temporomandibular Disorders (TMD) characterized by MRI-confirmed retrodiscal tissue hyperemia. Given the absence of a control group, this study represents a preliminary exploration of a clinical approach utilizing individualized interocclusal devices during infiltration. Methods: Twenty-eight patients (16 females, 12 males) with TMD and MRI evidence of retrodiscal tissue hyperemia were enrolled in this prospective, uncontrolled study. A unique protocol was employed, utilizing individualized interocclusal devices to optimize joint space access during bilateral ultrasound-guided infiltration of a mixture containing low-molecular-weight hyaluronic acid, lidocaine, and methylprednisolone acetate. Pain intensity (VAS 0–100 mm) and associated symptoms (tinnitus, vertigo, headache, joint clicking) were assessed at baseline and at 30, 60, and 90 days’ follow-up. Results: A statistically significant temporal reduction in pain was observed at all follow-up points (*p* < 0.001), with the mean VAS score decreasing from 70.5 ± 11.4 mm at baseline to 43.0 ± 11.1 mm at 90 days. Joint clicking disappeared in 80% of patients immediately after treatment. Conclusions: The ultrasound-guided infiltration technique, combined with personalized interocclusal support, demonstrated preliminary feasibility and short-term temporal improvement in pain and joint clicking in this specific patient cohort. Due to the lack of a control group and the multimodal nature of the intervention, these findings should be considered preliminary and do not allow for causal inferences regarding the efficacy of individual components.

## 1. Introduction

This study focused on patients who experienced pain in the preauricular region, both at rest and exacerbated during mastication, associated with an alteration of the condyle-disc complex dynamics, as an expression of an inflammatory process (inflammation) of the retrodiscal tissue. The purpose of this work was to evaluate the feasibility and temporal outcomes of the ultrasound-guided infiltration technique with hyaluronic acid and methylprednisolone in this specific patient population. The therapeutic choice was strictly guided by the MRI findings; the presence of retrodiscal tissue hyperemia provided a clear clinical rationale for a targeted intervention. By utilizing ultrasound guidance, it was possible to ensure that the injectate was delivered precisely into the area of inflammation identified by imaging, thereby maximizing the relevance of the treatment to the specific pathological state of the joint. This approach, already established in the treatment of other joint diseases [1,2,3], was applied here to the TMJ, intervening directly on the area of inflammation highlighted by MRI. Temporomandibular Disorders (TMDs) represent a complex of signs and symptoms affecting the temporomandibular joint (TMJ) and the masticatory musculature [4,5]. The scientific literature has extensively shown that patients with TMD present a heterogeneous symptomatology, which can include pain, functional limitations, joint noises, and otoneurological symptoms [5,6]. Recently, the therapeutic approach has been enriched with minimally invasive techniques, including intra-articular infiltrations, which have shown promising results in pain control and functional recovery [7,8]. The diagnosis of TMD is based on a careful clinical evaluation, which includes medical history, physical examination of the masticatory muscles, and analysis of mandibular dynamics, sometimes assisted by instrumental examinations such as mandibular kinesiography. The diagnostic imaging of choice for studying the joint components is dynamic Magnetic Resonance Imaging (MRI), which allows a detailed analysis of the morphology and position of the articular disc during mouth opening and closing movements, as well as the evaluation of the state of the soft tissues, such as the retrodiscal tissue, and the possible presence of joint effusion.

## 2. Materials and Methods

### 2.1. Study Design and Patient Selection

A prospective, uncontrolled pilot case series was conducted between January 2014 and December 2015 at the Center for Integrated Functional Orthodontics in Palermo. The study received approval from the local ethics committee (No. 1/2014, Ethics Committee of the University of Palermo) and was conducted in accordance with the principles of the Declaration of Helsinki. All participants provided written informed consent before enrollment.

Twenty-eight patients (16 females and 12 males), with a mean age of 39.1 (±8.9) years (range: 25–55), were selected. This study is presented as a preliminary pilot case series, and no a priori power analysis was performed. The inclusion period (2014–2015) reflects the initial implementation of this specific ultrasound-guided protocol; despite the delay in publication, the biomechanical principles and the use of individualized interocclusal devices remain relevant to current clinical practice discussions. The diagnosis was based on clinical examination and MRI findings; however, standardized Diagnostic Criteria for Temporomandibular Disorders (DC/TMD) were not strictly applied at the time of data collection, which is a limitation of this study. The use of MRI is supported by the literature highlighting its role in confirming clinical diagnoses of disc displacement, although the agreement between clinical criteria and imaging findings can vary [9].

Inclusion criteria were 1. clinical diagnosis of TMD based on medical history and physical examination; 2. presence of preauricular pain, assessed by VAS; 3. MRI evidence of altered signal in the retrodiscal tissue, indicative of inflammation.

Exclusion criteria were 1. history of maxillofacial trauma in the previous 6 months; 2. presence of fixed orthodontic treatments; 3. presence of complete removable dentures.

### 2.2. Preliminary Clinical and Instrumental Evaluation

The initial evaluation included the administration of a Visual Analogue Scale (VAS) to quantify the intensity of the pain perceived by the patient. Patients were also given a questionnaire to record the presence and characteristics of associated symptoms, as summarized in Table 1. The scale consisted of a 100 mm line, where 0 mm represented “no pain” and 100 mm “the worst imaginable pain”.

The primary endpoint was the temporal change in pain intensity (VAS 0–100 mm). Secondary outcomes included the qualitative evaluation of associated symptoms (headache, vertigo, tinnitus, and joint clicking) to descriptively characterize the study population.

### 2.3. Realization of Interocclusal Devices and MRI Acquisition

For each patient, impressions of the dental arches were taken in alginate, and the habitual occlusion and a position of mandibular protrusion were recorded using wax bites. Subsequently, in the dental laboratory, four interocclusal devices were made of silicone with a hardness of 63 shore A, with vertical thicknesses of 10, 20, 30, and 40 mm.

The patients were then sent to a radiology center for a dynamic MRI of the TMJ. A Philips Achieva 1.5 T D-stream machine, Milano, Italy was used. The MRI procedure was performed in a highly standardized manner to ensure diagnostic consistency and reproducibility. The acquisition protocol followed a rigorous sequence, including both static and dynamic evaluations with the mouth closed and at different degrees of opening using the calibrated interocclusal devices. This standardized approach allowed for a precise and objective assessment of the condyle-disc dynamics and the inflammatory state of the retrodiscal tissue across the entire study population.

### 2.4. Ultrasound-Guided Infiltration Procedure

All patients underwent a single intra-articular ultrasound-guided (US-guided) infiltration in both TMJs. The procedure was performed by an experienced operator (G.M., prepared for the procedure through extensive clinical practice) using a MyLab 70 XVG ultrasound machine (Esaote, Genova, Italy) with a multi-frequency linear probe (3–15 MHz), equipped with a needle guide.

**Figure 1:**
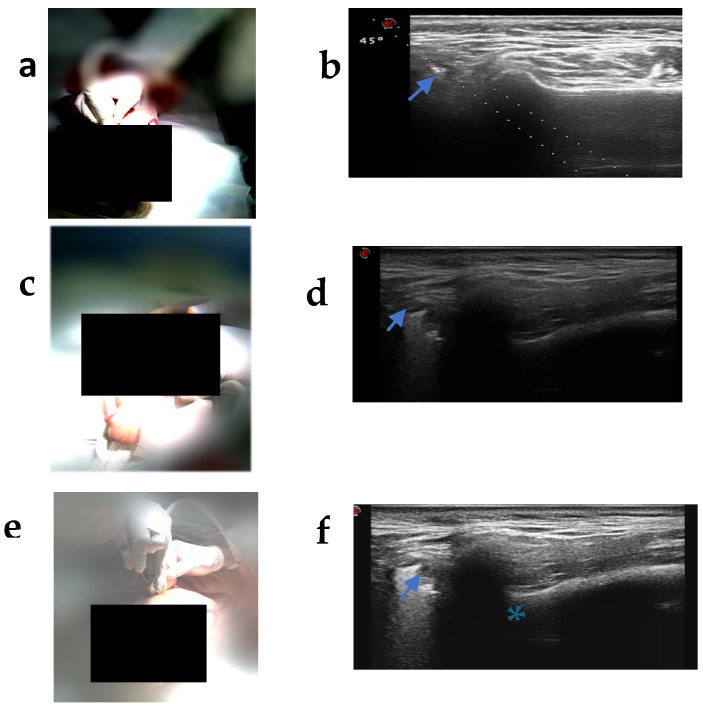
Ultrasound-guided intra-articular infiltration technique. (**a**) Positioning of the ultrasound probe in the preauricular region. (**b**) Pre-infiltration ultrasound image showing the needle guide at a 45° angle. (**c**) Needle insertion with an interocclusal device in place to ensure correct mandibular position. (**d**) Ultrasound image of the temporomandibular joint. (**e**) Infiltration in progress. (**f**) Ultrasound image during the infiltration procedure, showing the needle path. (Blue arrows: needle inside the joint; asterisk: mandibular condyle.)

The procedure was carried out as follows and is illustrated in Figure 1:

1. Patient positioning: the patient was positioned in lateral decubitus, contralateral to the side to be treated (Figure 1a).

2. Target identification: through longitudinal ultrasound scans with the mouth open and closed, the antero-superior portion of the joint capsule was identified as the target for the infiltration, in order to optimize the distribution of the drug in the bilaminar area (Figure 1b).

3. Preparation: After disinfecting the skin with a povidone–iodine solution, an interocclusal device (30 or 40 mm) was positioned to maintain the maximum possible opening and facilitate access to the glenoid fossa.

4. Anesthesia and Infiltration: After cutaneous anesthesia with ethyl chloride spray, a 20 G spinal needle was inserted with an oblique approach of 45° under continuous ultrasound guidance. Once the needle was positioned inside the capsule, 1 mL of low-molecular-weight hyaluronic acid (Sinovial HL 32, IBSA, Lodi, Italy) and 0.5 mL of a combined corticosteroid and anesthetic preparation (Depo-Medrol + Lidocaine, 40 mg/mL of methylprednisolone acetate and 10 mg/mL of lidocaine, Pfizer, New York, NY, USA) were injected. During the retraction of the needle, an additional 0.5 mL of methylprednisolone acetate was infiltrated in the periarticular area (Figure 1d).

The infiltration procedure was performed only after the construction of an individualized interocclusal device for each patient. This device served a critical dual purpose: first, it ensured the correct and stable opening required to optimize access to the retrocondylar tissue space under ultrasound guidance; second, it effectively prevented any involuntary mandibular movements by the patient during the procedure. This individualized stabilization was essential for maintaining the precision of the needle placement and ensuring the safety and efficacy of the intra-articular injection. The specific device thickness (30 or 40 mm) used during the infiltration procedure was individualized based on the patient’s maximum comfortable opening capacity and the optimal joint space visualization achieved during the preliminary ultrasound scan. This individualized approach aimed to maximize joint distraction and facilitate precise needle placement. All patients were treated with the selected device only during the infiltration procedure itself, not during the follow-up period.

### 2.5. Post-Infiltration Evaluation and Follow-Up

**Figure 2:**
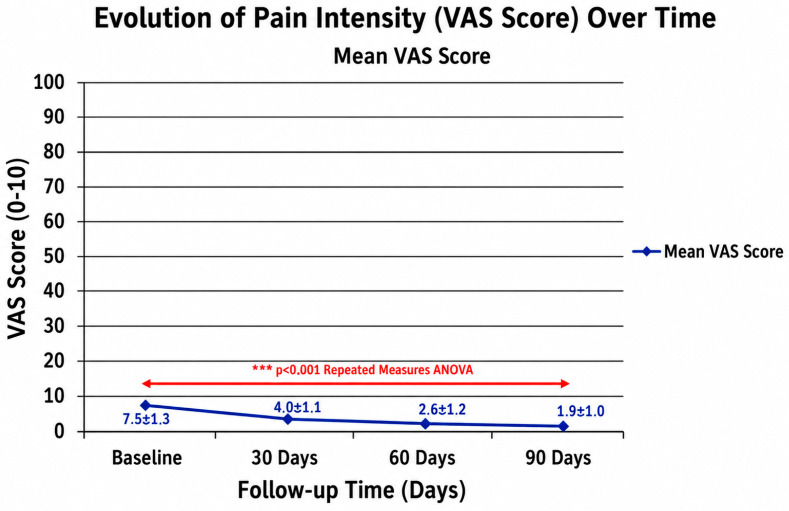
Evolution of pain intensity measured by Visual Analogue Scale (VAS) over the 90-day follow-up period. Data are presented as mean ± standard deviation. Repeated-measures ANOVA confirmed a statistically significant reduction in pain across all time points (F(3, 81) = 95.61, *** *p* < 0.001).

After the infiltration, the patients were monitored for 15 min. A dynamic ultrasound was performed to check the condylar sliding. The patients were re-evaluated clinically and with a control ultrasound at 24 h, 30 days, 60 days, and 90 days after the treatment to monitor the evolution of the symptomatology (Figure 2). The 24 h assessment was primarily aimed at evaluating immediate adverse events and acute pain relief.

### 2.6. Statistical Analysis

Statistical analysis was performed using SPSS software (version 25.0, IBM Corp., Armonk, NY, USA). Descriptive statistics were used to characterize the study population. The primary outcome (VAS score in mm) was analyzed per patient. Repeated-measures ANOVA was used to assess temporal changes in VAS scores. Assumptions of normality (Shapiro–Wilk test) and sphericity (Mauchly’s test) were tested. Mauchly’s test indicated that the assumption of sphericity had been violated (χ^2^(5) = 18.42, *p* = 0.002); therefore, degrees of freedom were corrected using Greenhouse–Geisser estimates (ε = 0.72). The F-statistic was reported as F(2.16, 58.32) = 95.61, *p* < 0.001. A *p*-value < 0.05 was considered statistically significant. Confidence intervals for the mean VAS scores were calculated at each time point. Effect sizes (Cohen’s d) for the reduction in VAS score from baseline were calculated using the following formula: d = (Mean1 − Mean2)/SD_pooled. The extremely large effect sizes (d > 2) likely reflect the uncontrolled nature of the study and the specific selection of patients with acute inflammatory findings. Given the preliminary nature of this pilot series, no control group was used, and results demonstrate temporal improvement rather than comparative efficacy. No post hoc corrections for multiple comparisons were applied, which is a limitation of this exploratory analysis.

## 3. Results

The study population consisted of 28 patients (16 females, 57.1%; 12 males, 42.9%) with a mean age of 39.1 ± 8.9 years. All patients completed the treatment protocol and the 90-day follow-up period.

### 3.1. Pain Assessment (VAS)

The mean pain score on the VAS (0–100 mm) at baseline (T0) was 70.5 ± 11.4 mm. After treatment, a statistically significant temporal reduction in pain was observed at all follow-up times, as confirmed by repeated-measures analysis of variance (ANOVA) (F(2.16, 58.32) = 95.61, *p* < 0.001). The mean scores are reported in Table 2.

### 3.2. Associated Symptoms

All 28 patients presented with joint clicking at baseline. Immediately after treatment, the disappearance of the joint click was observed in 80% of cases (22/28 patients). This improvement in joint mechanics was largely maintained throughout the 90-day follow-up period. Other investigated symptoms, such as headache, vertigo, and tinnitus, were subjectively reported as improved by the majority of patients; however, due to the lack of quantitative measurement using validated scales, these observations remain descriptive and should be interpreted with caution.

### 3.3. Adverse Events

The procedure was well tolerated by all patients. There was only one case (3.6%) of temporary and self-resolving paralysis of the mimic muscles ipsilateral to the injection site. The paresis resolved spontaneously within 4 h without any specific intervention. The mechanism is likely related to the inadvertent diffusion of the local anesthetic (lidocaine) towards the branches of the facial nerve, a known, albeit rare, complication of TMJ injections. No other complications, such as infection, persistent pain, or joint swelling, were reported during the follow-up period.

## 4. Discussion

The results of this preliminary pilot case series suggest that ultrasound-guided intra-articular infiltration with hyaluronic acid, lidocaine, and methylprednisolone is associated with a temporal reduction in pain symptoms and improvement in joint function in patients with TMD characterized by inflammation of the retrodiscal tissue. The reduction in pain (*p* < 0.001), observed as early as 30 days after treatment and maintained for up to 90 days, is an interesting finding in this specific cohort. These results are consistent with previous longitudinal observations, such as the one-year follow-up by Guarda-Nardini et al., which demonstrated the sustained effectiveness of HA injections in patients with TMJ osteoarthritis [10,11]. Furthermore, the efficacy of this approach has been documented across different age groups, including elderly patients, highlighting its broad clinical applicability [12]. However, due to the lack of a control group and the multimodal nature of the intervention, these results demonstrate temporal improvement rather than definitive therapeutic efficacy, and causal inferences cannot be made [13,14].

This study, although conducted between 2014 and 2015, provides insights into a personalized approach to TMD management. The delay in publication is attributed to the long-term observation of the clinical implementation of this protocol. While advancements in ultrasonography have occurred, the fundamental biomechanical principles and the use of individualized interocclusal devices remain pertinent to current clinical practice discussions [15]. The precision offered by ultrasound guidance aims to ensure accurate placement of the injectate, potentially minimizing risks [16,17]. This approach is consistent with the evolution toward minimally invasive techniques, such as the single-needle arthrocentesis protocol proposed by Guarda-Nardini et al., which aims to simplify the procedure while maintaining clinical efficacy [10]. The transparency regarding the study’s age is crucial, as contemporary standards for TMD diagnosis and treatment have evolved since 2015.

The hypothesized mechanism of action is multifaceted: lidocaine provides immediate pain relief, methylprednisolone addresses the acute inflammatory process in the retrodiscal tissue, and hyaluronic acid improves joint lubrication and disc mobility [18,19,20]. The use of individualized interocclusal devices during the procedure may further facilitate joint distraction and needle access [16]. However, the combined administration of these components prevents the attribution of the observed effects to any single intervention, representing a significant confounding factor in this study design.

Although not directly investigated, the potential influence of the craniocervical–mandibular region’s biomechanical relationships, such as the tongue–mandible–hyoid system [12], and conditions like Eagle syndrome [21] on TMJ function warrants further investigation. The precision of ultrasound-guided techniques, as demonstrated in this study, could be valuable in exploring these complex craniofacial disorders in future research.

Several limitations must be acknowledged. First, the absence of a control group, randomization, and blinding prevents the assessment of comparative efficacy and causal inference. Second, the sample size (n = 28) is relatively small, limiting the generalizability of the findings. Third, the multimodal nature of the intervention (HA, lidocaine, corticosteroids, and interocclusal devices) makes it impossible to isolate the effect of each component. Fourth, the lack of standardized DC/TMD criteria and the qualitative assessment of secondary outcomes further limit the reproducibility and interpretability of the results. Finally, the long delay between data collection and publication may affect the contemporary relevance of the findings in the context of rapidly evolving TMJ treatment methods.

## 5. Conclusions

The ultrasound-guided infiltration technique, combined with personalized interocclusal support, demonstrated preliminary feasibility and short-term temporal improvement in pain and joint clicking in this specific patient cohort. These findings suggest that a personalized, ultrasound-guided approach may be a viable option for managing TMD symptoms associated with retrodiscal inflammation. However, given the significant methodological limitations, including the lack of a control group and the multimodal intervention, these results must be interpreted with caution. Future randomized controlled trials with larger sample sizes and standardized diagnostic criteria are necessary to establish the definitive efficacy and safety of this protocol.

## Figures and Tables

**Table 1:** Questionnaire for the evaluation of symptoms associated with TMD.

Symptom	Characteristic	Options
Headache	Frequency	(1) Upon waking, (2) Afternoon, (3) Evening
	Localization	Frontal, Occipital, Parietal, Temporal
Vertigo	Type	(1) Subjective, (2) Objective
Tinnitus	Laterality	(1) Unilateral, (2) Bilateral
Cervical ROM	Evaluation	(1) Regular, (2) Limited
Dysphagia	Presence	(1) Present, (2) Absent
Neck Tension	Presence/Laterality	(1) Present (Unilateral/Bilateral), (2) Absent
Joint Clicking	Presence	(1) Present, (2) Absent

**Table 2:** Evolution of the VAS score (mm) over time.

Time Point	Mean VAS Score (±SD)	% Reduction vs. T0	*p*-Value	95% CI
Baseline (T0)	70.5 (±11.4)	-	-	(66.1, 75.0)
30 days (T1)	48.3 (±10.0)	31.5%	<0.001	(44.4, 52.2)
60 days (T2)	44.5 (±9.4)	37.0%	<0.001	(40.8, 48.1)
90 days (T3)	43.0 (±11.1)	39.0%	<0.001	(38.7, 47.4)

## Data Availability

The data presented in this study are available on request from the corresponding author. The data are not publicly available due to privacy and ethical restrictions.
